# Group B streptococcus is the most common pathogen for septic arthritis with unique clinical characteristics: data from 12 years retrospective cohort study

**DOI:** 10.1186/s41927-019-0084-5

**Published:** 2019-09-16

**Authors:** Rungkan Ruksasakul, Pongthorn Narongroeknawin, Paijit Assavatanabodee, Sumapa Chaiamnuay

**Affiliations:** Rheumatic Disease Unit, Department of Internal Medicine, Phramongkutklao Hospital and College of Medicine, 315 Ratchawithi road, Bangkok, Ratchathewi District 10400 Thailand

**Keywords:** Bacterial septic arthritis, Group B streptococcus, Oligoarthritis, Polyarthritis, Tenosynovitis, Rainy season

## Abstract

**Background:**

Group B Streptococcus (GBS) emerged as the frequent pathogen for septic arthritis. There was no study comparing risks, clinical presentations and outcomes between GBS septic arthritis and other bacterial septic arthritis.

The aim of this study is to evaluate the differences in risks, clinical presentations, and outcomes of GBS septic arthritis and other bacterial septic arthritis, and identify independent risks and clinical presentations suggesting GBS septic arthritis.

**Method:**

Medical records of patients diagnosed with non-gonococcal bacterial arthritis admitted in Phramongkutklao Hospital during 2006–2018 were reviewed. Associated risks, clinical presentations and outcomes were compared between GBS septic arthritis (GBS group) and other bacterial septic arthritis (other bacterial group).

**Result:**

Two hundred and thirty one cases of non-gonococcal bacterial arthritis confirmed by positive joint fluid cultures and/or hemocultures were included. The three most common pathogens were GBS (37.7%), *Staphylococcus aureus* (23.4%) and *Streptococcus viridans* (7.4%). GBS group was more commonly found in rainy season than other bacterial group. Patients in GBS group were less likely to have underlying diseases and had more number of involved joints than those in other bacterial group. The clinical presentations more commonly found in GBS group than other bacterial group were oligo-polyarthritis, upper extremities joint involvement, axial joint involvement, tenosynovitis and central nervous system involvement.

Multivariate analysis found the independent associated factors of GBS arthritis are tenosynovitis, oligo-polyarthritis and rainy season.

**Conclusions:**

GBS is now the most common pathogen for bacterial septic arthritis. The independent associated factors of GBS arthritis were oligo-polyarthritis, tenosynovitis and rainy season.

**Electronic supplementary material:**

The online version of this article (10.1186/s41927-019-0084-5) contains supplementary material, which is available to authorized users.

## Background

Bacterial septic arthritis is one of the emergency rheumatic conditions because of serious morbidities, permanent disabilities, and death with mortality rates of 10–15% [1–3]. Delayed or inadequate treatment can lead to irreversible joint destruction and morbidities which occur in about 25–50% of affected patients [2–4]. The estimated incidence of bacterial septic arthritis varies from 4 to 10 per 100,000 people per year [2, 5–7]. The main causative pathogens for septic arthritis are *Staphylococcus aureus* and Streptococcus species [8–10].

Group B Streptococcus (GBS) is recognized as a cause of sepsis and meningitis in newborns and pregnant women [11, 12]. Several population-based surveys of bacteremia have raised concerns about the growing incidence of GBS disease in non-pregnant adults [13–18]. Clinical manifestations in adults include skin and soft tissue infections, urinary tract infections, endocarditis, pneumonia, meningitis, peritonitis and osteoarticular manifestations [19, 20]. Recently, previous studies have shown that the incidence of septic arthritis has changed. GBS has emerged as the main cause of bacterial arthritis in adults [10, 21] and some studies showed GBS was the most common pathogen of bacterial septic arthritis [22].

Louthrenoo W, et al. reported that the average age of GBS septic arthritis patients was quite young at 52.9 years old. Most patients were not pregnant or it did not occur during the peripartum period. Most cases occurred in rainy and early winter seasons. Two-thirds of the patients had at least one underlying disease. GBS septic arthritis patients often had characteristic oligoarticular or polyarticular arthritis and involvement of the small joints which differed from bacterial septic arthritis caused by other pathogens. Furthermore, GBS septic arthritis involved uncommon area (example spine involvement) and had cellulitis over and adjacent to infected joints [21]. However, there has been no prior study comparing risks, clinical presentations, and outcomes between GBS septic arthritis and other bacterial septic arthritis.

The objectives of this study were to distinguish risks, clinical features, and outcomes of GBS septic arthritis from other septic arthritis.

## Methods

Medical records of patients aged 18 years old and above who were diagnosed with non-gonococcal bacterial septic arthritis in Phramongkutklao Hospital during 2006–2017 were retrospectively reviewed.

**Fig. 1:**
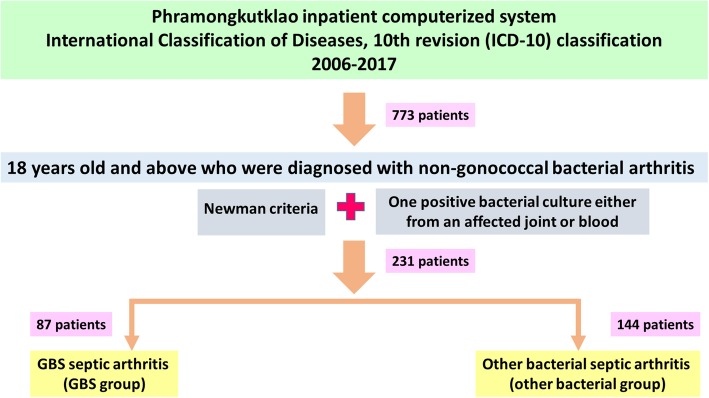
The flowchart of the study

Patients were identified from Phramongkutklao inpatient computerized system according to the International Classification of Diseases, 10th revision (ICD-10) classification. Coding included bacterial septic arthritis as a principal diagnosis, a comorbidity or a complication during the admission. Codes starting with M00 and M01 referred to forms of pyogenic arthritis and direct infections of the joint, respectively. Patients were included if non-gonococcal bacterial septic arthritis was diagnosed by Newman criteria [23] plus they had at least one positive bacterial culture either from an affected joint or blood. The flowchart of this study was depicted in Fig. 1.

Non-gonococcal bacterial septic arthritis was diagnosed by Newman criteria [23] and required at least one of four points to be met:

1. Isolation of a pathogenic organism from an affected joint
2. Isolation of a pathogenic organism from another source (e.g. blood) in the context of a hot red joint suspicious of sepsis
3. Typical clinical features and turbid joint fluid in the presence of previous antibiotic treatment
4. Postmortem or pathological features suspicious of septic arthritis

Patients with prosthetic joint infection and gonococcal bacterial septic arthritis were excluded.

Baseline characteristics, clinical presentations, laboratory findings, cause of infection, treatment and outcomes were thoroughly reviewed. Associated risks, clinical presentations and outcomes were compared between GBS septic arthritis (GBS group) and other bacterial septic arthritis (other bacterial group).

Definitions of variables collected in this study are as follows;

1. Diabetes mellitus was diagnosed if patients met any of the following criteria [24]
  1. Fasting plasma glucose > 126 mg/dL (7.0 mmol/L).

Fasting was defined as no caloric intake for at least 8 h.

2) Two-hour plasma glucose > 200 mg/dL (11.1 mmol/L) during an OGTT.

The test should be performed as described by the WHO, using a glucose load containing the equivalent of 75 g anhydrous glucose dissolved in water.

3) Hemoglobin A1C > 6.5% (48 mmol/mol).

The test should be performed in a laboratory using a method that is NGSP certified and standardized to the DCCT assay.

4) In a patient with classic symptoms of hyperglycemia or hyperglycemic crisis, a random plasma glucose > 200 mg/dL (11.1 mmol/L).

2. Liver disease defined as chronic hepatitis B, C infection or cirrhosis [25]
3. End-stage renal disease defined as patients with glomerular filtration rate (GFR) < 15 ml/min/1.73m2 [26]
4. Season was categorized according to the Thai Meteorological Department [27]
  - Rainy season: 16 May – 15 October
  - Winter season: 16 October – 13 February
  - Summer season: 14 February – 15 May
5. Complications defined as serious medical events occurred after the admission for bacterial septic arthritis including sepsis, shock, intubation, admission into an intensive care unit, pneumonia, upper urinary tract infection, pressure sore, and osteomyelitis.
6. Upper joint included sternoclavicular joints, acromioclavicular joints, shoulder joints, elbow joints, wrist joints, metacarpophalangeal joints and proximal interphalangeal joints.
7. Axial joint included sternoclavicular joints, acromioclavicular joints, shoulder joints, sacroiliac joint, hip joints and spine.
8. Small joint included metacarpophalangeal joints, proximal interphalangeal joints, metatarsophalangeal joints, wrist and interphalangeal joints of toes [28].

Conventional biochemical test was used for bacterial identification. The growth obtained was identified by colony morphology, Gram-stain of the isolated colonies, and conventional biochemical identification tests as per the standard protocol followed in our laboratory. GBS identification was confirmed by positive CAMP (Christie, Atkins, Munch-Petersen) test.

From the previous study, the prevalence of GBS septic arthritis in the study population was 15.7% [21]. In order to determine the point prevalence within a 4.7% margin of error assuming a confidence level of 95% a sample size of 230 was required [29].

### Statistical analyses

Data were analyzed with SPSS software (Statistical Package for the Social Sciences, version 22, Chicago, IL, USA). Continuous data and categorical data were presented as mean ± standard deviation (SD) or median and interquartile range (IQR) and percent, respectively. The baseline characteristics, clinical presentations, laboratory findings and treatment outcomes were compared between the GBS group and other bacterial group. Parametric statistics (Independent sample T-test and Chi-square test) were used if data were normally distributed. Nonparametric statistics (Mann Whitney-U test and Fisher’s exact test) were used if any of data were skewed. Odds ratios (OR) with 95% confidence interval (CI) were calculated to identify risks and clinical characteristics between the GBS group and other bacterial group. All tests were two-tailed tests with *p* < 0.05. Risk factors associated with the GBS septic arthritis were assessed by multiple logistic regression models. Variables adjusted in multiple logistic regression models were clinically relevant variables or variables with *p*-value < 0.1 from the univariate analyses.

## Results

Two hundred and thirty one cases of non-gonococcal bacterial septic arthritis confirmed by positive joint fluid cultures and/or hemocultures diagnosed in Phramongkutklao Hospital from January 2006 – December 2017 were included. Two-thirds of patients were male. The mean age ± SD was 60.8 ± 17.3 years old. The five most common pathogens were GBS *(*37.7%), *Staphylococcus aureus* (23.4%), Viridans group *Streptococcus* (7.4%), *Salmonella* spp. (5.6%) and Group A *Streptococcus* (4.3%). The causative pathogens for septic arthritis were described in Additional file 1.

There were 87 patients in the GBS group and 144 patients in the other bacterial group. No significant differences in age, gender, and body mass index (BMI) were found between both groups.

Bacterial septic arthritis was more commonly found in rainy season in both groups with more common in GBS septic arthritis than other bacterial septic arthritis (71.3% vs 38.2%; *p* < 0.001). Comorbidities were more commonly found in the other bacterial group than the GBS group (86.8% vs 66.7%, *p* < 0.001) such as diabetic mellitus (38.2% vs 18.4%, *p* = 0.002) and liver disease (18.1% vs 4.6%, *p* = 0.003).

Furthermore, the other bacterial group commonly had previous history of joint infection (4.9% vs 0%, *p* = 0.047), skin infection (20.1% vs 1.1%, *p* < 0.001) and intra-articular steroid injection (4.9% vs 0%, *p* = 0.047) than in the GBS group. The demographics and clinical characteristics in the GBS and other bacterial septic arthritis group were depicted in Table 1.

**Table 1:** The demographics and clinical characteristics in GBS and other bacterial septic arthritis group

Variable	All (231)	GBS group (87)	Other bacterial group (144)	*p-value*
Female (n, %)	86, 37.2%	36, 41.4%	50, 34.7%	0.311
Age mean: (mean ± SD)	60.8 + 17.3	58.6 + 16.1	62.2 + 18.0	0.136
Body Mass Index: Median (IQR 25-IQR75)	23.4 (20.8–25.8)	23.0 (21.1–25.0)	23.4 (20.8–26.4)	0.608
Season (n, %)				
Summer	47, 20.3%	11, 12.6%	36, 25.0%	0.024
Rainy	130, 56.3%	62, 71.3%	68, 47.2%	< 0.001
Winter	54, 23.4%	14,16.1%	40, 27.8%	0.042
Underlying disease (n, %)	183, 79.2%	58, 66.7%	125, 86.8%	< 0.001
Rheumatoid arthritis	5, 2.2%	1, 1.1%	4, 2.8%	0.653
Diabetic mellitus	71, 30.7%	16, 18.4%	55, 38.2%	0.002
ESRD (n, %)	12, 5.2%	2, 2.3%	10, 6.9%	0.219
Liver disease	30, 13.0%	4, 4.6%	26, 18.1%	0.003
Malignancy	28, 12.1%	9, 10.3%	19, 13.2%	0.520
Hypertension	110, 47.6%	40, 46.0%	70, 48.6%	0.698
Hyperlipidemia	77, 33.3%	30, 34.5%	47, 32.6%	0.773
History of joint infection (n, %)	7, 3.0%	0, 0%	7, 4.9%	0.047
History skin infection (n, %)	30, 13.0%	1, 1.1%	29, 20.1%	< 0.001
History of joint surgery (n, %)	4, 1.7%	1, 1.1%	3, 2.1%	1.000
History of intraarticular steroid injection (n, %)	7, 3.0%	0, 0%	7, 4.9%	0.047
Current medication (n, %)				
Prednisolone	20, 8.7%	4, 4.6%	16, 11.1%	0.088
Chemotherapy	8, 3.5%	2, 2.3%	6, 4.2%	0.363
Immunosuppressive drug	9, 3.9%	2, 2.3%	7, 4.9%	0.489

*p* < 0.05 defined statistical significance, *IQR* Interquartile range, *ESRD* End stage renal disease

Rainy season: 16 May – 15 October, Winter season:16 October – 13 February, Summer season:14 February – 15 May

There was no difference in the mean ± SD of duration from the onset of symptom to hospital admission between two groups (5 days [IQR25-IQR75:2–10] in the GBS group and 4 days [IQR25-IQR75:2–7] in the other bacterial group). Patients in the GBS group had more number of involved joints than those in the other bacterial group with a median of 3 joints (IQR25-IQR75:1–5) and 1 joint (IQR25-IQR75:1–2); *p* < 0.001, respectively. The clinical presentations more commonly found in the GBS group than the other bacterial group were oligo-polyarthritis (72.4% vs 31.9%, *p* < 0.001), upper extremity joint involvement (71.2% vs 35.4%, *p* < 0.001), axial joint involvement (37.9% vs 19.4%, *p* = 0.002), tenosynovitis (39.1% vs 2.1%, *p* < 0.001) and central nervous system (CNS) involvement (10.3% vs 2.8%, *p* = 0.03). Whereas patients in the the other bacterial group were more likely to present with monoarthritis (68.1% vs 27.6%, *p* < 0.001), more concomitant skin infection (16.7% vs 6.9%, *p* = 0.032) and more infections in other organs (36.8% vs 4.6%, *p* < 0.001) than patients in the GBS group. CNS involvement in the GBS group included meningitis, meningoencephalitis, brain abscess and epidural abscess. CNS involvement in the other bacterial group included meningitis and meningoencephalitis. Three patients in the GBS group had bacterial endophthalmitis, but the other bacterial group had no ocular involvement.

Knee was the most common affected joint in both groups (64.4% in the GBS group and 62.5% in other bacterial group, *p* = 0.775). Ankle (34.5% vs 20.1%, *p* = 0.015), shoulder (33.3% vs 20.1%, *p* = 0.025), wrist (31.0% vs 9.7%, *p* < 0.001), sternoclavicular (SC) (21.8 vs 6.3, *p* < 0.001), elbow (17.2% vs 4.9%, *p* < 0.002), spine (13.8% vs 3.5%, *p* = 0.004), and metacarpophalangeal (MCP) (13.8% vs 0.7%, *p* < 0.001), proximal interphalangeal (PIP) (13.8% vs 1.4%, *p* < 0.001) and interphalangeal (IP) joints of toe (4.6% vs 0%, *p* = 0.019) were more commonly affected in the GBS group than the other bacterial group. The details of the differences in clinical presentations of GBS septic arthritis and other bacterial septic arthritis were described in Table 2.

**Table 2:** The clinical presentations in GBS and other bacterial septic arthritis group

Variable	All (231)	GBS group (87)	Other bacterial group (144)	*p-value*
Symptom duration: Median (IQR25-IQR75)	5.0 (2.0–10.0)	5.0 (2.0–10.0)	4.0 (2.0–7.0)	0.655
Initial temperature: Median (IQR25-IQR75)	37.8 (37.0–38.4)	37.9 (37.2–38.5)	37.8 (37.0–38.3)	0.301
Number of joint involvement: Median (IQR25-IQR75)	1.0 (1.0–3.0)	3.0 (1.0–5.0)	1.0 (1.0–2.0)	< 0.001
Pattern of joint involvement (n, %)				
Monoarthritis	122, 52.8%	24, 27.6%	98, 68.1%	< 0.001
Oligoarthritis	58, 25.1%	27, 31.0%	31, 21.5%	0.106
Polyarthritis	51, 22.1%	36, 41.4%	15, 10.4%	< 0.001
Upper joint involvement	113, 48.9%	62, 71.3%	51, 35.4%	< 0.001
Axial joint involvement	61, 26.4%	33, 37.9%	28, 19.4%	0.002
Small joint involvement	22, 9.5%	9, 10.3%	13, 9.0%	0.741
Site of joint involvement (n, %)				
Knee	146, 63.2%	56, 64.4%	90, 62.5%	0.775
Ankle	59, 25.5%	30, 34.5%	29, 20.1%	0.015
Shoulder	58, 25.1%	29, 33.3%	29, 20.1%	0.025
Wrist	41, 17.7%	27, 31.0%	14, 9.7%	< 0.001
Sternoclavicular	28, 12.1%	19, 21.8	9, 6.3%	< 0.001
Elbow	22, 9.5%	15, 17.2%	7, 4.9%	0.002
Spine	17, 7.4%	12, 13.8%	5, 3.5%	0.004
Site of joint involvement (n, %)				
MCP	13, 5.6%	12, 13.8%	1, 0.7%	< 0.001
PIP	14, 6.1%	12, 13.8%	2, 1.4%	< 0.001
Hip	25, 10.8%	11, 12.6%	14, 9.7%	0.489
AC	13, 5.6%	8, 9.2%	5, 3.5%	0.081
MTP	16, 6.9%	8, 9.2%	8, 5.6%	0.291
Interphalangeal of toe	4, 1.7%	4, 4.6%	0, 0%	0.019
Sacroiliac	5, 2.2%	2, 2.3%	3, 2.1%	0.624
Extra articular involvement (n, %)				
Tenosynovitis	37, 16.0%	34, 39.1%	3, 2.08%	< 0.001
CNS	13, 5.7%	9, 10.3%	4, 2.8%	0.035
Skin involvement	30, 13.0%	6, 6.9%	24, 16.7%	0.032
Tendon rupture	5, 2.2%	4, 4.6%	1, 0.7%	0.068
Ocular involvement	3, 1.3%	3, 3.4%	0, 0%	0.052
Cardiac involvement	6, 2.6%	1, 1.1%	5, 3.5%	0.414
Concomitant infection (n, %)	57, 24.7%	4, 4.6%	53, 36.8%	< 0.001

*p* < 0.05 defined statistical significance, IQR: Interquartile range, symptom duration: Duration from the onset of joint symptoms to the hospital admission, *IQR* Interquartile range, *SC* Sternoclavicular, *MCP* Metacarpophalangeal, *PIP* Proximal interphalangeal, *AC* Acromioclavicular, *MTP* Metatarsophalangeal, *CNS* Central nervous system, GBS Streptococcus group B

Upper joint included sternoclavicular joints, acromioclavicular joints, shoulder joints, elbow joints, wrist joints, metacarpophalangeal joints and proximal interphalangeal joints

Axial joint included sternoclavicular joints, acromioclavicular joints, shoulder joints, sacroiliac joint, hip joints and spine

Small joint included metacarpophalangeal joints, proximal interphalangeal joints, metatarsophalangeal joints,wrist and interphalangeal joints of toes

Multivariate analyses examined the joints involvement that were independently associated with GBS septic arthritis (adjusted for knee, ankle, shoulder, wrist, SC, elbow, spine, MCP, PIP, hip, AC, MTP, IP of toe and SC joints) were MCP, PIP, spine and SC joints with adjusted OR 16.2, 7.0, 4.6 and 3.2 with 95% CI of 1.9–137.2, 1.3–36.0, 1.4–14.9 and 1.2–8.2, respectively. Data were described in Table 3.

**Table 3:** Multivariate analyses of factors associated with the GBS septic arthritis

Parameter	All (231)	GBS group (87)	Other bacterial group (144)	Adjust odds	95% CI	*p*-value
Associated joints^a^						
MCP (n, %)	13, 5.6%	12, 13.8%	1, 0.7%	16.2	1.9–137.2	0.011
PIP (n, %)	14, 6.1%	12, 13.8%	2, 1.4%	7.0	1.3–36.0	0.021
Spine (n, %)	17, 7.4%	12, 13.8%	5, 3.5%	4.6	1.4–14.9	0.011
SC (n, %)	28, 12.1%	19, 21.8%	9, 6.3%	3.2	1.2–8.2	0.017
Associated factors^b^						
Tenosynovitis (n, %)	37, 16.0%	34, 39.08%	3, 2.08%	21.0	5.5–79.6	< 0.001
Rainy season (n, %)	130, 56.3%	62, 71.3%	68, 47.2%	3.6	1.8–7.5	< 0.001
Oligo-polyarthritis (n, %)	109, 47.2%	63, 72.4%	46, 31.9%	2.6	1.3–5.2	0.008
Associated laboratory data^c^						
Baseline hemoglobin (mg/dL): (mean ± SD)	11.0 + 2.2	11.7 + 2.4	10.5 + 2.1	1.3	1.1–1.5	< 0.001

*p* < 0.05 defined statistical significance,

*GBS* Streptococcus group B, *MCP* Metacarpophalangeal, *PIP* Proximal interphalangeal, *SC* Sternoclavicular

^a^Adjusted for knee, ankle, shoulder, wrist, SC, elbow, spine, MCP, PIP, hip, AC, MTP, IP of toe and SI joints

^b^Adjusted for age, gender, rainy season, body mass index, diabetic mellitus, end-stage renal disease, liver disease, oligo-polyarthritis, upper joint involvement, tenosynovitis, tendon rupture, ocular involvement and central nervous system involvement

^c^Adjusted for baseline serum WBC, hemoglobin, creatinine, hs-CRP, AST and ALT

White blood cell count in synovial fluid was not different in both groups (54,000.0 *cells*/cu.mm. [IQR25-IQR75: 25,600.0-131,000.0] in the GBS group and 52,800.0 *cells*/cu.mm. [IQR25-IQR75: 24,030.0-106,000.0], *p* = 0.640 in the other bacterial group). However, the GBS group showed higher peripheral white blood cell count than the other bacterial group (15,000.0 *cells*/cu.mm. [IQR25-IQR75: 10,700.0-19,900.0] vs 12,500.0 *cells*/cu.mm. [IQR25-IQR75: 8550.0-16,950.0], *p* = 0.029) and higher level of high sensitivity C-reactive protein (hs-CRP) (226.9 mg/L [IQR25-IQR75: 142.2–300.0] vs 157.0 mg/L [IQR25-IQR75: 85.3–267.9], *p* = 0.030).

Median initial blood urea nitrogen (BUN) and serum creatinine (sCr) levels were more elevated in the other bacterial group than the GBS group (BUN 24.4 mg/dl [IQR25-IQR75: 14.5–43.6] vs 18.4 mg/dl [IQR25-IQR75: 12.9–35.1], *p* = 0.040 and sCr 1.2 mg/dl [IQR25-IQR75: 0.8–2.5] vs 0.9 mg/dl [IQR25-IQR75: 0.8–1.5], *p* = 0.033). Furthermore, overall patients in the other bacterial group had lower mean hemoglobin level than the GBS group (10.5 + 2.1 vs 11.7 + 2.4 mg/dL; *p* < 0.001).

Percentage of positive blood and synovial fluid cultures were comparable in the GBS group and the other bacterial group (71.3% vs 66.0% in hemoculture, *p* = 0.404; 63.2% vs 69.4% in synovial fluid culture, *p* = 0.329). Laboratory data were described in Table 4.

**Table 4:** The laboratory profiles in GBS and other bacterial septic arthritis group

Variable	All (231)	GBS group (87)	Other bacterial group (144)	*p-value*
WBC in synovial fluid (*cells*/cu.mm.): Median (IQR25-IQR75)	53,000.0 (25,200.0-110,720.0)	54,000.0 (25,600.0-131,000.0)	52,800.0 (24,030.0-106,000.0)	0.640
Hb (mg/dL): Mean ± SD	11.0 + 2.2	11.7 + 2.4	10.5 + 2.1	< 0.001
Serum WBC (*cells*/cu.mm.): Median (IQR25-IQR75)	13,400.0 (9500.0-18,800.0)	15,000.0 (10,700.0-19,900.0)	12,500.0 (8550.0-16,950.0)	0.029
BUN (mg/dL): Median (IQR25-IQR75)	21.8 (13.8–39.6)	18.4 (12.9–35.1)	24.4 (14.5–43.6)	0.040
Cr (mg/dL): Median (IQR25-IQR75)	1.0 (0.8–2.2)	0.90 (0.8–1.5)	1.20 (0.8–2.5)	0.033
AST (IU/L): Median (IQR25-IQR75)	39.0 (26.5–69.8)	37.0 (25.0–59.0)	42.0 (27.0–71.0)	0.182
ALT (IU/L): Median (IQR25-IQR75)	33.5 (20.0–55.5)	35.0 (24.0–59.0)	32.0 (17.0–54.0)	0.175
ESR (min/hr): Median (IQR25-IQR75)	109.0 (86.0–119.0)	104.5 (85.0–118.0)	111.0 (86.0–119.0)	0.558
hs-CRP (mg/L): Median (IQR25-IQR75)	185.2 (100.8–300.0)	226.9 (142.2–300.0)	157.0 (85.3–267.9)	0.030
Positive hemoculture (n, %)	157, 68%	62, 71.3%	95, 66.0%	0.404
Positive synovial fluid culture (n, %)	155, 67.1%	55, 63.2%	100, 69.4%	0.329

*p* < 0.05 defined statistical significance, *IQR* Interquartile range, *WBC* White blood cell, *Hb* Hemoglobin, *BUN* Blood urea nitrogen, *AST* Aspartate aminotransferase, *ALT* Alanine Aminotransferase, *ESR* Erythrocyte sedimentation rate, *hs-CRP* High Sensitivity C-Reactive Protein, *GBS* Streptococcus group B

No significant differences were found between the GBS group and the other bacterial group according to the rate of complications (49.4% vs 61.8%, *p* = 0.065*)*, the surgical rate (62.1% vs 55.6%, *p =* 0.331) and the length of stay (27.0 days [IQR25-IQR75: 19.0–38.0] vs 31.0 days [IQR25-IQR75: 18.0–47.0], *p* = 0.378). However, the mortality rate was significantly decreased in the GBS group compared with the other bacterial group (5.74% vs 22.91%, *p* < 0.001). There were no differences in the median duration of total antibiotics treatment and intravenous antibiotic course between the GBS group and the other bacterial group (60 days [IQR25-IQR75: 42.0–143.0) vs 56 days (IQR25-IQR75: 42.0–102.0], *p* = 0.287) and 28 days [IQR25-IQR75: 19.0–37.0] vs 30 days [IQR25-IQR75: 21.0–45.0], *p* = 0.134). The treatment outcomes in the GBS and other bacterial septic arthritis groups were shown in Table 5.

**Table 5:** The treatment outcomes in GBS and other bacterial septic arthritis group

Variable	All (231)	GBS group (87)	Other bacterial group (144)	*P* value
Surgery (n, %)	134, 58.0%	54, 62.1%	80, 55.6%	0.331
Complication (n, %)	132, 57.1%	43, 49.4%	89, 61.8%	0.065
Death (n, %)	38, 16.5%	5, 5.7%	33, 22.9%	< 0.001
Period of antibiotic treatment				
Total course (days): Median (IQR25-IQR75)	57.0 (42.0–109.0)	60.0 (42.0–143.0)	56.0 (42.0–102.0)	0.287
Intravenous form (days): Median (IQR25-IQR75)	29.0 (21.0–42.0)	28.0 (19.0–37.0)	30.0 (21.0–45.0)	0.134
Oral form (days): Median (IQR25-IQR75)	30.0 (8.0–76.0)	34.5 (17.0–98.0)	28.0 (0.0–60.0)	0.007
Length of stay (days): Median (IQR25-IQR75)	29.0 (18.0–42.0)	27.0 (19.0–38.0)	31.0 (18.0–47.0)	0.378

*p* < 0.05 defined statistical significance, *IQR* Interquartile range, *GBS* Streptococcus group B. Complications included sepsis, shock, intubation, admit to intensive unit care, pneumonia, upper urinary tract infection, pressure sore and osteomyelitis

Multivariate analyses adjusted for age, gender, season, BMI, diabetic mellitus, end-stage renal disease, liver disease, oligo-polyarthritis, upper joint involvement, tenosynovitis, tendon rupture, ocular involvement and central nervous system involvement were performed. The independently associated factors of GBS arthritis were tenosynovitis, oligo-polyarthritis and rainy season with ORs 21.0, 2.6, 3.6 and 95% confidence intervals of 5.5–79.6, 1.3–5.2 and 1.8–7.5, respectively.

We analyzed factors associated with mortality and found that the death group was more likely to have underlying diseases (92.1% vs 76.7%, *p* = 0.030*)*, end stage renal disease (13.2% vs 3.6%, *p* = 0.031*)*, and hypertension (68.4% vs 43.5%, *p* = 0.007*)*, than the survival group. The death group was more likely to have concomitant infection (39.5% vs 21.8%, *p* = 0.025*)*, positive synovial fluid culture (86.8% vs 63.2%, *p* = 0.004*)* and positive hemoculture (80.0% vs 65.8%, *p* = 0.130) compared to the survival group. The death group was likely to have GBS septic arthritis (5.7% vs 94.3%, *p* < 0.001) and more likely to have *Staphylococcus aureus* septic arthritis (31.5% vs 68.5%, *p* = 0.001). The multivariate analysis found that hypertension at baseline, positive hemoculture and synovial fluid culture were associated with death, whereas, GBS septic arthritis was less likely to be associated with death with the adjusted OR and 95% CI of 2.63 (1.12–5.86), 2.95 (1.18–7.38), 5.67 (1.94–16.58), and 0.21 (0.07–0.59), respectively. Data were shown in Table 6.

**Table 6:** The demographics and clinical characteristics in death and survived patients and factor associated with death

Variable (n, %)	All (*n* = 231)	Univariate analyses			Multivariate analyses^a^	
		Death (*n* = 38)	Survived (*n* = 193)	*p*-value	Adjusted Odd ratio (95% confidence interval)	*p*-value
Underlying disease	183, 79.2%	35, 92.1%	148, 76.7%	.030	1.01 (0.23–4.51)	0. 981
End staged renal disease	12, 5.2%	5, 13.2%	7, 3.6%	.031	3.53 (0.91–13.73)	0.069
Hypertension	110, 47.6%	26, 68.4%	84, 43.5%	.007	2.63 (1.12–5.86)	0.047
Axial joint involvement	61, 26.4%	5, 13.2%	56, 29.0%	.045	0.45 (0.16–1.32)	0.156
Concomitant infection	57, 24.7%	15, 39.5%	42, 21.8%	.025	1.57 (0.67–3.69)	0.343
Positive synovial fluid culture	155, 67.1%	33, 86.8%	122, 63.2%	0.004	5.67 (1.94–16.58)	0.002
Positive hemoculture	157, 68.0%	30, 80.0%	127, 65.8%	0.130	2.95 (1.18–7.38)	0.020
Group B streptococcus	87, 37.7%	5, 5.7%	82, 94.3%	< 0.001	0.21 (0.07–0.59)	0.003
*Staphylococcus aureus*	54, 23.4%	17, 31.5%	37, 68.5%	0.001	1.34 (0.55–3.29)	0.515

^a^adjusted for age, sex, Group B streptococcus, *Staphylococcus aureus* group, Underlying disease, Hypertension, End stage renal disease, axial joint involvement, concomitant infection, positive synovial fluid culture and positive hemoculture

## Discussion

*Staphylococcus aureus* was previously recognized as the most common pathogen that caused bacterial septic arthritis in adults [5, 30–32]. Two studies of septic arthritis from 1976 to 1995 reported in 1996 found that the incidence of GBS arthritis was uncommon (1–3%) [5, 33]. However, recent studies found that the causative pathogens had changed. The study from France between 1979 and 1998 in 303 bacterial septic arthritis patients reported that the frequency of streptococci septic arthritis slightly increased over time [10]. The study from Thailand reported 38 cases with GBS septic arthritis from July 1990 to December 2010 in which almost 90% of the cases were seen between 2008 and 2010, thus reflecting that GBS had become an emerging cause of septic arthritis [21]. However, the most common pathogen which still caused bacterial septic arthritis was *Staphylococcus aureus* in both studies. Our study reviewed the bacterial septic arthritis during 2006 to 2017 and it is the first study which reported that GBS had now become the most common pathogen for bacterial septic arthritis (37.7%), while *Staphylococcus aureus* was the second most common causative pathogen (23.4%). The clinical characteristics of *Staphylococcus aureus* septic arthritis are similar to what has been described in previous studies [4, 5, 30]. Data was depicted in Additional file 2: Table S2.

GBS has long been recognized as a causative pathogen of infection in newborns and pregnant women [1]. Recently, GBS has been recognized as an ever-growing cause of serious invasive infections in non-pregnant adults [18, 19]. This study reviewed that age and gender risk factors were similar between GBS patients and other bacterial septic arthritis patients.

GBS septic arthritis in our study was more commonly found in rainy season. This finding was similar to the previous studies from Chiangmai University, Thailand which found that 97.1% of GBS septic arthritis occurred between May and November [21] and invasive GBS infection most commonly occurred in September [34]. Seasonal variation of GBS infection was also described in other parts of the world. The study from Iran revealed that invasive GBS infection most commonly occurred in moist and cold weather in December, January and August [35]. A recent report from active, population-based surveillance in 10 US sites participating in the Active Bacterial Core Surveillance/Emerging Infections Program Network also found invasive GBS infections in non-pregnant adults are more prevalent in a late summer [36]. Reasons for seasonal variability of invasive GBS infections are unclear, but some possibilities include environmental conditions such as moist weather which could promote the growth and spread of GBS. In the US, the reasons proposed for the late summer peak of invasive GBS infections in non-pregnant adults were that there might be factors increasing risks of skin and soft tissue infections and less likely, increased exposure to bovine *S. agalactiae* strains in summer months. GBS has been linked to bovine mastitis and can be isolated from milk samples obtained in mastitis control programs. However, distinct subtypes, clonal groups and host specificities among human and bovine strains of GBS suggest a very low likelihood for cross species transmission. Further studies from the other parts of the world are warranted to confirm this hypothesis.

Comorbid diseases were common in bacterial septic arthritis patients, but when compared between GBS and other bacterial groups, the comorbid diseases such as diabetes mellitus and liver disease were more commonly found in the other bacterial than the GBS group.

GBS septic arthritis had unique clinical characteristics compared with other bacterial septic arthritis including more number of joint involvements which were more likely (70%) to be oligo-polyarthritis. The majority of patients in other bacterial septic arthritis in our study were affected in one joint as monoarthritis. The knee was the most commonly affected joint in both groups. Upper extremities and axial joint involvements were more common in GBS septic arthritis than other bacterial septic arthritis. These findings were consistent with previous reports [2, 5, 10, 21, 37–39]; although none of these studies had compared GBS arthritis with other bacterial septic arthritis.

The multivariate regression model found that the distinctive joints associated with GBS septic arthritis rather than other bacterial septic arthritis were MCPs, PIPs, spine and SC joint. Thus if septic arthritis was suspected in these joints, it would suggest that GBS might be the causative pathogen.

Our study is the first report that found tenosynovitis was extremely common in GBS septic arthritis (39.1%) which has seldomly presented before in other bacterial septic arthritis (2.1%). The multivariate analyses found that tenosynovitis had an adjusted OR 21.0 and 95% CI 5.5–79.6 for predicting GBS septic arthritis. Previous reports of GBS septic arthritis found concomitant cellulitis was common [10, 21]; although our report found that cellulitis was found in 6.9% in the GBS septic arthritis group which was less common than the other bacterial group (16.7%).

CNS infection and pan-ophthalmitis were previously found in 18.4 and 2.6% of GBS septic arthritis patients [21]. Our study found that CNS and ocular infections were commonly found in GBS septic arthritis more than other bacterial septic arthritis (10.3% vs 2.8 and 3.4% vs 0%, respectively).

Laboratory data found that GBS septic arthritis seemed to be quite different from the other bacterial septic arthritis such as higher peripheral white blood cell count and higher hs-CRP levels. However, lower hemoglobin, higher serum BUN, and creatinine levels were observed in the other bacterial group than the GBS group. There were no differences in rates of positive blood and synovial fluid cultures between the two groups, which were similar to previous studies [10, 21].

Mortality rate was higher in the other bacterial group than the GBS group. The rates of surgery and complications were comparable in both groups. The duration of oral antibiotics was longer in the GBS group than the other bacterial group, which could be due to more spinal involvement that required an extended duration of oral antibiotics in the GBS group.

There were several limitations in our study. Firstly, this was a retrospective study. Some data such as radiographic outcomes were not performed and recorded in a standardized manner, thus we did not include radiographic outcomes in this analysis. However, there was less than 1% missing data. Secondly, septic arthritis patients were divided into only two groups which were GBS septic arthritis (GBS group) and other bacterial septic arthritis. In the other bacterial group was the combination of various bacterial pathogens, which might have their own characteristics in terms of risk factors, clinical presentations, and outcomes. Thirdly, this study was performed in a tertiary care academic center in Bangkok (central part of Thailand) and this might not represent characteristics of bacterial septic arthritis in other countries. Nonetheless, our data was very consistent with previous reports from Chiangmai University (Northern part of Thailand) [21, 34].

## Conclusions

This is the first report which demonstrated that GBS has become the most common pathogen for bacterial septic arthritis. GBS septic arthritis usually presented with oligo-polyarthritis and tenosynovitis. Upper extremity and axial joint involvements were more common in GBS septic arthritis. GBS septic arthritis frequently occurred in the rainy season. More incidence of CNS infection, less co-morbidities, and lower mortality rates in GBS septic arthritis were evident compared with other bacterial septic arthritis.

## Additional files

Additional file 1: The causative pathogens for bacterial septic arthritis**.** The causative pathogens for bacterial septic arthritis from joint isolates or hemoculture and death separated by each organism. Descriptive data of the pathogens for bacterial septic arthritis in this cohort. (DOCX 16 kb)

Additional file 2: Characteristics of *Staphylococcus aureus* septic arthritis. The demographics, clinical characteristics and outcome in *Staphylococcus aureus* and other bacterial septic arthritis. The demographics, clinical characteristics and outcome in *Staphylococcus aureus* compared to other bacterial septic arthritis. (DOCX 15 kb)

## Data Availability

The data that support the findings of this study are available on request from the corresponding author.

## Abbreviations

AC: Acromioclavicular
BMI: Body mass index
BUN: Blood urea nitrogen
CI: Confidence interval
GBS: Group B streptococcus
GFR: Glomerular filtration rate
IP: Interphalangeal
IQR: Interquartile range
MCP: Metacarpophalangeal
mg/dL: Milligrams Per Deciliter
mmol/L: Millimoles Per Liter
MTP: Metatarsophalangeal
OR: Odds ratios
PIP: Proximal interphalangeal
SC: Sternoclavicular
sCr: Serum creatinine
SD: Standard deviation

## References

[1] Kaandorp CJ, Krijnen P, Moens HJ, Habbema JD, van Schaardenburg D (1997). The outcome of bacterial arthritis: a prospective community-based study. Arthritis Rheum.

[2] Weston VC, Jones AC, Bradbury N, Fawthrop F, Doherty M (1999). Clinical features and outcome of septic arthritis in a single UK Health District 1982-1991. Ann Rheum Dis.

[3] Mathews CJ, Weston VC, Jones A, Field M, Coakley G (2010). Bacterial septic arthritis in adults. Lancet..

[4] Goldenberg DL (1998). Septic arthritis. Lancet..

[5] Morgan DS, Fisher D, Merianos A, Currie BJ (1996). An 18 year clinical review of septic arthritis from tropical Australia. Epidemiol Infect.

[6] Geirsson AJ, Statkevicius S, Vikingsson A (2008). Septic arthritis in Iceland 1990-2002: increasing incidence due to iatrogenic infections. Ann Rheum Dis.

[7] Rutherford AI, Subesinghe S, Bharucha T, Ibrahim F, Kleymann A, Galloway JB (2016). A population study of the reported incidence of native joint septic arthritis in the United Kingdom between 1998 and 2013. Rheumatology (Oxford).

[8] Daynes J, Roth MF, Zekaj M, Hudson I, Pearson C, Vaidya R (2016). Adult native septic arthritis in an Inner City hospital: effects on length of stay. Orthopedics..

[9] Gupta MN, Sturrock RD, Field M (2001). A prospective 2-year study of 75 patients with adult-onset septic arthritis. Rheumatology (Oxford).

[10] Dubost JJ, Soubrier M, De Champs C, Ristori JM, Sauvezie B (2004). Streptococcal septic arthritis in adults. A study of 55 cases with a literature review. Joint Bone Spine.

[11] Zangwill KM, Schuchat A, Wenger JD (1992). Group B streptococcal disease in the United States, 1990: report from a multistate active surveillance system. MMWR CDC Surveill Summ.

[12] Madhi SA, Radebe K, Crewe-Brown H, Frasch CE, Arakere G, Mokhachane M (2003). High burden of invasive Streptococcus agalactiae disease in south African infants. Ann Trop Paediatr.

[13] Farley MM, Harvey RC, Stull T, Smith JD, Schuchat A, Wenger JD (1993). A population-based assessment of invasive disease due to group B Streptococcus in nonpregnant adults. N Engl J Med.

[14] Jackson LA, Hilsdon R, Farley MM, Harrison LH, Reingold AL, Plikaytis BD (1995). Risk factors for group B streptococcal disease in adults. Ann Intern Med.

[15] Blumberg HM, Stephens DS, Modansky M, Erwin M, Elliot J, Facklam RR (1996). Invasive group B streptococcal disease: the emergence of serotype V. J Infect Dis.

[16] Munoz P, Llancaqueo A, Rodriguez-Creixems M, Pelaez T, Martin L, Bouza E (1997). Group B streptococcus bacteremia in nonpregnant adults. Arch Intern Med.

[17] Ko WC, Lee HC, Wang LR, Lee CT, Liu AJ, Wu JJ (2001). Serotyping and antimicrobial susceptibility of group B Streptococcus over an eight-year period in southern Taiwan. Eur J Clin Microbiol Infect Dis.

[18] Blancas D, Santin M, Olmo M, Alcaide F, Carratala J, Gudiol F (2004). Group B streptococcal disease in nonpregnant adults: incidence, clinical characteristics, and outcome. Eur J Clin Microbiol Infect Dis.

[19] Farley MM (2001). Group B streptococcal disease in nonpregnant adults. Clin Infect Dis.

[20] Falagas ME, Rosmarakis ES, Avramopoulos I, Vakalis N (2006). *Streptococcus agalactiae* infections in non-pregnant adults: single center experience of a growing clinical problem. Med Sci Monit.

[21] Louthrenoo W, Kasitanon N, Wangkaew S, Hongsongkiat S, Sukitawut W, Wichainun R (2014). Streptococcus agalactiae: an emerging cause of septic arthritis. J Clin Rheumatol.

[22] Paosong S, Narongroeknawin P, Pakchotanon R, Asavatanabodee P, Chaiamnuay S (2015). Serum procalcitonin as a diagnostic aid in patients with acute bacterial septic arthritis. Int J Rheum Dis.

[23] Newman JH (1976). Review of septic arthritis throughout the antibiotic era. Ann Rheum Dis.

[24] Chobanian AV, Bakris GL, Black HR (2003). The seventh report of the joint national committee on prevention, detection, evaluation, and treatment of high blood pressure: the jnc 7 report. JAMA..

[25] Ginès P, Angeli P, Lenz K, Møller S, Moore K, Moreau R, et al. EASL clinical practice guidelines on the management of ascites, spontaneous bacterial peritonitis, and hepatorenal syndrome in cirrhosis. J Hepatol. 2010;53(3):397–417. 10.1016/j.jhep.2010.05.004 20633946

[26] Levey AS, Coresh J (2012). Chronic kidney disease. Lancet.

[27] Meteorological Department of Thailand [Internet]. Bangkok, Thailand, 2015. The Climate of Thailand; 2015 [cited 2019 Jan 1]; [about 7 screens]. Available from: https://www.tmd.go.th/en/archive/thailand_climate.pdf

[28] Aletaha D, Neogi T, Silman AJ, Funovits J, Felson DT, Bingham CO (2010). 2010 rheumatoid arthritis classification criteria: an American College of Rheumatology/European league against rheumatism collaborative initiative. Arthritis Rheum.

[29] Wayne WD. Biostatistics: A foundation of analysis in the health sciences. 6, editor. United States of America: Wiley; 1995. p. 180.

[30] Goldenberg DL, Reed JI (1985). Bacterial arthritis. N Engl J Med.

[31] Le Dantec L, Maury F, Flipo RM, Laskri S, Cortet B, Duquesnoy B (1996). Peripheral pyogenic arthritis. A study of one hundred seventy-nine cases. Rev Rhum Engl Ed.

[32] Ryan MJ, Kavanagh R, Wall PG, Hazleman BL (1997). Bacterial joint infections in England and Wales: analysis of bacterial isolates over a four year period. Br J Rheumatol.

[33] Osiri M, Akkasilpa S, Reinprayoon S, Deesomchok U (1996). Streptococcal arthritis in Thai adults: case series and review. J Med Assoc Thai.

[34] Chaiwarith R, Jullaket W, Bunchoo M, Nuntachit N, Sirisanthana T, Supparatpinyo K (2011). *Streptococcus agalactiae* in adults at Chiang Mai University Hospital: a retrospective study. BMC Infect Dis.

[35] Shayanfar N, Mohammadpour M, Hashemi-Moghadam SA, Ashtiani MT, Mirzaie AZ, Rezaei N (2012). Group B streptococci urine isolates and their antimicrobial susceptibility profiles in a group of Iranian females: prevalence and seasonal variations. Acta Clin Croat.

[36] Phares CR, Lynfield R, Farley MM, Mohle-Boetani J, Harrison LH, Petit S (2008). Epidemiology of invasive group B streptococcal disease in the United States, 1999-2005. Jama..

[37] Nolla JM, Gomez-Vaquero C, Corbella X, Ordonez S, Garcia-Gomez C, Perez A (2003). Group B streptococcus (Streptococcus agalactiae) pyogenic arthritis in nonpregnant adults. Medicine (Baltimore).

[38] Alejandro Balsa EM-M. Rheumatology. 6, editor. Philadelphia: Elsevier Ltd; 2014. p 887.

[39] Ravindran V, Logan I, Bourke BE (2009). Medical vs surgical treatment for the native joint in septic arthritis: a 6-year, single UK academic Centre experience. Rheumatology (Oxford).

