# Supplementary material for: Group B streptococcus is the most common pathogen for septic arthritis with unique clinical characteristics: data from 12 years retrospective cohort study
Source: BMC Rheumatol. 2019 Sep 16;3:38. doi: 10.1186/s41927-019-0084-5 (PMC6745788; doi:10.1186/s41927-019-0084-5)
Supplement: Supplementary file 1 — The causative pathogens for bacterial septic arthritis. The causative pathogens for bacterial septic arthritis from joint isolates or hemoculture and death separated by each organism. Descriptive data of the pathogens for bacterial septic arthritis in this cohort. (DOCX 16 kb) [file 41927_2019_84_MOESM1_ESM.docx]

**Additional file 1: Table S1. The causative pathogens for bacterial septic arthritis from joint isolates or hemoculture and death separated by each organism**

| Organism | Death, n  (n=38) | Total  (n=231) |
| --- | --- | --- |
| *Acinetobacter baumannii* | 1 | 1 |
| *Proteus mirabilis* | 1 | 1 |
| *Bacillus* spp. | 1 | 1 |
| *Klebsiella pneumoniae* | 2 | 5 |
| *Enterobacter* spp. | 1 | 3 |
| *Staphylococcus aureus* | 17 | 54 |
| *Escherichia coli* | 3 | 10 |
| *Streptococcus pyogenase* | 3 | 10 |
| *Burkholderia pseodomallei* | 1 | 5 |
| *Pseudomonas aeruginosa* | 1 | 6 |
| *Salmonella* spp. | 1 | 13 |
| Viridans group Streptococci | 1 | 17 |
| Group B *Streptococcus* | 5 | 87 |
| *Streptococcus bovis* | 0 | 5 |
| *Campylobacter* spp. | 0 | 1 |
| *Streptobacillus* spp. | 0 | 1 |
| *Corynebacterium* spp. | 0 | 2 |
| *Enterococcus* spp. | 0 | 4 |
| *Aeromonas* spp. | 0 | 2 |
| *Streptococcus pneumoniae* | 0 | 2 |
| *Streptococcus suis* | 0 | 1 |
|  |  |  |
