# Supplementary material for: Group B streptococcus is the most common pathogen for septic arthritis with unique clinical characteristics: data from 12 years retrospective cohort study
Source: BMC Rheumatol. 2019 Sep 16;3:38. doi: 10.1186/s41927-019-0084-5 (PMC6745788; doi:10.1186/s41927-019-0084-5)
Supplement: Supplementary file 2 — Characteristics of Staphylococcus aureus septic arthritis. The demographics, clinical characteristics and outcome in Staphylococcus aureus and other bacterial septic arthritis. The demographics, clinical characteristics and outcome in Staphylococcus aureus compared to other bacterial septic arthritis. (DOCX 15 kb) [file 41927_2019_84_MOESM2_ESM.docx]

**Additional file 2: TableS2 The demographics, clinical characteristics and outcome in Staphylococcus aureus and other bacterial septic arthritis**

| **Variable** | **All**  **(231)** | **Staph**  **(54)** | **Other bacterial group**  **(177)** | ***p-value*** |
| --- | --- | --- | --- | --- |
| Female (n, %) | 86, 37.2% | 13, 24.1% | 73, 41.2% | .025 |
| Age mean: (mean±SD) | 60.8+17.3 | 63.45+16.79 | 60.0+16.79 | 0.381 |
| Season (n, %) |  |  |  |  |
| Summer | 47, 20.3% | 12, 22.2% | 35, 19.8% | 0.869 |
| Rainy | 130, 56.3% | 25, 46.3% | 82, 46.3% | 1.00 |
| Winter | 54, 23.4% | 12, 22.2% | 35, 19.8% | 0.702 |
| Diabetic mellitus | 71, 30.7% | 23, 42.6% | 48, 27.1% | 0.043 |
| ESRD (n, %) | 12, 5.2% | 8, 14.8% | 4, 2.3% | 0.001 |
| Liver disease | 30, 13.0% | 14, 25.9% | 16, 9.0% | 0.002 |
| History skin infection (n, %) | 30, 13.0% | 16, 29.6% | 14, 7.9% | <0.001 |
| History of intraarticular steroid injection (n, %) | 7, 3.0% | 5, 9.3% | 2, 1.1% | 0.009 |
| Pattern of joint involvement (n, %) | | | | |
| Monoarthritis | 122, 52.8% | 37, 68.5% | 85, 48.0% | 0.009 |
| Oligoarthritis | 58, 25.1% | 13, 24.1% | 58, 32.8% | 0.243 |
| Polyarthritis | 51, 22.1% | 4, 7.4% | 34, 19.2% | 0.057 |
| Extra articular involvement (n, %) | | | | |
| Skin involvement | 30, 13.0% | 12, 22.2% | 18, 10.2% | 0.035 |
| Cardiac involvement | 6, 2.6% | 4, 7.4% | 2, 1.1% | 0.028 |
| Death (n, %) | 38, 16.5% | 17, 31.5% | 21, 11.9% | 0.001 |
